# Impact of Conservative Versus Conventional Oxygenation on Outcomes of Patients in Intensive Care Units: A Systematic Review and Meta-analysis

**DOI:** 10.7759/cureus.5662

**Published:** 2019-09-15

**Authors:** Takashi Hirase, Eric S Ruff, Iqbal Ratnani, Salim R Surani

**Affiliations:** 1 Orthopedics and Sports Medicine, Houston Methodist Hospital, Houston, USA; 2 Plastic Surgery, University of Texas Medical Branch, Galveston, USA; 3 Anesthesiology and Critical Care, Houston Methodist Hospital, Houston, USA; 4 Internal Medicine, Texas A&M Health Science Center, Temple, USA

**Keywords:** hyperoxia, mechanical ventilation, intensive care unit

## Abstract

Background: There is mixed evidence in the superiority of conservative versus conventional approach to oxygen therapy among patients admitted into the intensive care unit (ICU). The purpose of this study was to determine if conservative versus conventional oxygenation results in a statistically significant difference in outcomes in ICU patients.

Methods: A systematic review was registered with the International Prospective Register of Systematic Reviews (PROSPERO) and performed using Preferred Reporting Items for Systematic Reviews and Meta-Analyses (PRISMA) guidelines. Inclusion criteria consisted of Level I-IV investigations of conservative versus conventional oxygenation among ICU patients. ICU mortality, 28-day mortality, in-hospital mortality, ICU length-of-stay, hospital length-of-stay, rate of new infections, and rate of new non-respiratory organ failure were compared using two-sample Z-tests using p-value less than 0.05.

Results: Three thousand four hundred thirty-three articles were screened. Four articles were included in the analysis. Three hundred seventy-two patients under the conservative oxygenation arm (Minimum target SpO2: 88-94%) and 370 patients under the conventional oxygenation arm (Minimum target SpO2: 96-97%) were analyzed. ICU mortality (16.7 ± 9.5% vs. 22.7 ± 6.0%; P<0.01), 28-day mortality (34.6 ± 26.4% vs. 41.6 ± 14.6%; P=0.02), and in-hospital mortality (30.2 ± 22.5% vs. 37.7 ± 14.2%; P<0.01) were all significantly lower in the conservative oxygenation arm versus the conventional oxygenation arm, respectively. Rate of new non-respiratory organ failure was also significantly lower in the conservative oxygenation arm (20.0 ± 8.5% vs. 29.7 ± 11.7%; P<0.01).

Conclusion: The authors conclude that conservative oxygenation therapy could result in significantly lower rates of ICU mortality, 28-day mortality, in-hospital mortality, and new-onset non-respiratory organ failure. Further randomized controlled studies that show clinical outcome improvement in multiple parameters may be worthwhile to assess the true efficacy of this practice.

## Introduction

Oxygen supplementation is often used universally to counteract acute hypoxemia in a hospital setting. Despite the widespread strategy of providing oxygenation therapy in critically ill patients, there are currently no explicit target values for arterial oxyhemoglobin saturation (SpO_2_) [1]. However, studies have demonstrated that inattentiveness to SpO_2_ levels while providing oxygenation therapy may lead to periods of hyperoxemia and tissue hyperoxia that may result in atelectasis, interstitial fibrosis, and tracheobronchitis through the induction of alveolar protein leakage and neutrophil infiltration [2-5]. High mortality rates and other adverse outcomes have also been associated with hyperoxemia among critically ill patients [6-9]. Despite numerous evidence of adverse effects, patients continue to experience prolonged episodes of hyperoxemia in the clinical setting [10].

Conservative oxygenation therapy to minimize harmful effects from hyperoxemia have been used successfully in patients with acute respiratory distress syndrome (ARDS) as well as in acutely ill patients [11-15]. However, particularly in critically ill patients, concerns from adverse effects from hypoxemia may supersede that of hyperoxemia [16-18]. Due to such opposing risks, there are conflicting opinions and evidence on conservative versus conventional oxygenation therapy, particularly among patients in the intensive care unit (ICU). 

Current studies evaluating the outcomes of conservative versus conventional oxygenation therapy in the ICU are limited to a few prospective randomized controlled studies. Thus, the purpose of this investigation was to determine if conservative oxygenation versus conventional oxygenation results in a statistically significant difference in outcomes in ICU patients. We hypothesized that patients receiving conservative oxygenation therapy would have significantly superior outcomes compared to patients receiving conventional oxygenation therapy. 

## Materials and methods

**Figure 1:**
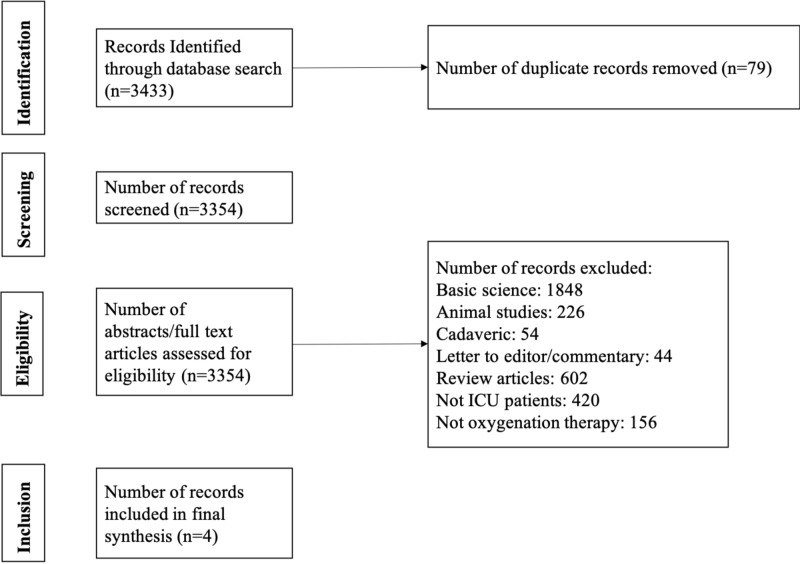
Flow diagram summarizing the literature search, screening, and review

A systematic review was registered with PROSPERO on February 15, 2018 (ID: CRD42018088872). PRISMA guidelines were followed [19]. Inclusion criteria consisted of Level I-IV (via Oxford Centre for Evidence-Based Medicine [CEBM]) therapeutic studies that investigated conservative versus conventional oxygenation among ICU patients [20]. Studies that included non-ICU patients were excluded. Basic science and animal studies, cadaveric studies, expert opinions, letters to editors, and review articles were excluded. Studies published in non-English languages were not excluded but were unidentified in the medical databases. In the event of different studies with duplicate subject populations, the study with the longer follow-up, higher level of evidence, a greater number of subjects, or greater clarity of methods and results was included. The authors conducted separate searches of the following medical databases: MEDLINE, Web of Science, and Cochrane Central Register of Controlled Trials databases. Under the PROSPERO registration, similar prior systematic reviews and meta-analyses were sought, and none were identified. The searches were performed on February 16, 2018. The search terms were “conservative oxygenation”, “conventional oxygenation”, and “intensive care unit”. The search results were reviewed for duplicates and the inclusion criteria to determine articles that were included in the final analysis (Figure 1).

Two authors independently reviewed all articles. The study design, patient populations, and intervention technique were first identified. All outcome measures and complication rates were analyzed. The levels of evidence were then assigned based on the CEBM [20]. The Modified Coleman Methodology Score (MCMS) was used to analyze study methodological quality [21]. Only the outcome measurements used by more than 50% of the studies were included in the data analysis to increase the power of the measurement over that of individual studies. Weighted means of values under conservative and conventional oxygenation arms from each study were calculated, and comparisons were made using two-sample Z-tests using a p-value of less than 0.05 for significance. ICU mortality, 28-day mortality, in-hospital mortality, ICU length-of-stay, hospital length-of-stay, rate of new infections, and rate of new non-respiratory organ failure were compared. Conservative oxygenation was defined as a minimum SpO2 target of 88-94%. Conventional oxygenation was defined as a minimum SpO2 target of 96-97%.

## Results

Three thousand four hundred thirty-three articles were screened (Figure 1). Four articles were included in the analysis (Table 1) [22-25]. All four articles were level II evidence. According to MCMS, all four articles were good (scores between 70 and 84) [21]. The mean MCMS was 81.5 ± 1.9. There were 742 patients analyzed. Three hundred seventy-two patients were under the conservative oxygenation arm (Minimum target SpO2: 88-94%) and 370 patients under the conventional oxygenation arm (Minimum target SpO2: 96-97%). Two hundred and fourteen males and 158 females were under the conservative oxygenation arm, and 230 males and 140 females were under the conventional oxygenation arm (P=0.20). The mean ages were 62.2 ± 3.9 years and 64.1 ± 3.5 years under the conservative and conventional oxygenation arms, respectively (P<0.01). The mean Acute Physiology and Chronic Health Evaluation (APACHE) III scores were 86.7 ± 30.3 and 87.4 ± 32.4 under the conservative and conventional oxygenation arms, respectively (P=0.85). Two hundred ninety-nine patients (80.4%) under the conservative oxygenation arm and 300 patients (81.1%) under the conventional oxygenation arms were mechanically ventilated at the initiation of each study (P=0.81). 

**Table 1:** Study demographics. NR-Not recorded, APACHE-Acute Physiology and Chronic Health Evaluation, LOS-length of stay, MV-mechanical ventilation, CA-cardiac arrest

Study		Suzuki et al. 2014 [24]	Panwar et al. 2015 [22]	Girardis et al. 2016 [25]	Eastwood et al. 2016 [23]	Weighted mean, SD	P-value
Type of Study		Prospective, randomized controlled study	Prospective, randomized controlled study	Prospective, randomized controlled study	Prospective, randomized controlled study	N/A	N/A
Level of Evidence		II	II	II	II	N/A	N/A
Included Patients		Age ≥ 18 admitted to ICU on MV with expected MV time ≥ 48 hours	Age ≥ 18 admitted to ICU on MV with expected MV time ≥ 24 hours	Age ≥ 18 admitted to ICU with expected ICU LOS ≥ 72 hours	Age ≥ 18 admitted to ICU on MV post CA		
SpO_2_ Minimum Target							
	Conservative	90%	88%	94%	88%	91.8 ± 2.8	< 0.01
	Conventional	97%	96%	97%	97%	96.9 ± 0.5	
No. patients							
	Conservative	54	52	216	50	147.2 ± 82.0	0.73
	Conventional	51	51	218	50	149.3 ± 83.7	
Age, SD (IQR)							
	Conservative	56 ± 16	62.4 ± 14.9	63 (51-74)	65 (59-77)	62.2 ± 3.9	< 0.01
	Conventional	59 ± 17	62.4 ± 17.4	65 (52-76)	67 (50-71)	64.1± 3.5	
Female gender (%)							
	Conservative	22 (40.7%)	20 (38.5%)	95 (44.0%)	21 (42.0%)	42.5%	0.20
	Conventional	13 (25.5%)	18 (35.3%)	93 (42.7%)	16 (32.0%)	37.8%	
Mechanical Ventilation N, (%)							
	Conservative	54 (100%)	52 (100%)	143 (66.2%)	50 (100%)	80.4%	0.81
	Conventional	51 (100%)	51 (100%)	148 (67.9%)	50 (100%)	81.1%	
APACHE III score (IQR)							
	Conservative	62 (49-92)	79.5 (61-92.5)	NR	121 (105-142)	86.7 ± 30.3	0.85
	Conventional	68 (42-94)	70 (50-84)	NR	125 (107-141)	97.4 ± 32.35	

ICU mortality (16.7 ± 9.5% vs. 22.7 ± 6.0%; P <0.01), 28-day mortality (34.6 ± 26.4% vs. 41.6 ± 14.6%; P=0.02), and in-hospital mortality (30.2 ± 22.5% vs. 37.7 ± 14.2%; P<0.01) were all significantly lower in the conservative oxygenation arm versus the conventional oxygenation arm, respectively (Tables 2, 3). Rate of new non-respiratory organ failure was also significantly lower in the conservative oxygenation arm (20.0 ± 8.5% vs. 29.7 ± 11.7%; P<0.01). There were no significant differences in ICU LOS (6.2 ± 2.5 days vs. 6.0 ± 1.0 days; P=0.19), hospital LOS (18.9 ± 6.7 days vs. 18.3 ± 6.0 days; P=0.23) and new onset infections (26.0 ± 27.8% vs. 29.0 ± 22.6%; P=0.17) between conservative and conventional oxygenation arms, respectively. 

**Table 2:** Individual Study Outcomes Measures. NR-Not recorded, ICU-intensive care unit, LOS-length-of-stay

Study		Suzuki et al. 2014 [24]	Panwar et al. 2015 [22]	Girardis et al. 2016 [25]	Eastwood et al. 2016 [23]
ICU Mortality, N (%)					
	Conservative	NR	13 (25%)	25 (11.6%)	15 (30%)
	Conventional	NR	12 (24%)	44 (20.2%)	16 (32%)
28-d mortality, N (%)					
	Conservative	9 (16.7%)	NR	NR	27 (54%)
	Conventional	16 (31.4%)	NR	NR	26 (52%)
Hospital mortality, N (%)					
	Conservative	NR	NR	52 (24.2%)	28 (56%)
	Conventional	NR	NR	74 (33.9%)	27 (54%)
ICU LOS, Days (IQR)					
	Conservative	NR	9 (5-13)	6 (4-10)	4 (2-7)
	Conventional	NR	7 (4-12)	6 (4-11)	5 (4-9)
Hospital LOS, Days (IQR)					
	Conservative	NR	20 (10-25)	21 (13-38)	9 (3-17)
	Conventional	NR	16 (7-30)	21 (12-34)	9 (4-24)
New infections, N (%)					
	Conservative	31 (57.4)	NR	39 (18.1)	NR
	Conventional	28 (54.9)	NR	50 (22.9)	NR
New non-respiratory organ failure, N (%)					
	Conservative	16 (29.6)	NR	38 (17.6)	NR
	Conventional	22 (43.1)	NR	58 (26.6)	NR

**Table 3:** Average Study Outcome Measures Included in Best Evidence Synthesis. ICU-intensive care unit, LOS-length of stay

	ICU Mortality (%)	28-day Mortality (%)	In-hospital Mortality (%)	ICU LOS (Days)	Hospital LOS (Days)	New Infections (%)	New non-respiratory organ failure (%)
Conservative	16.7 ± 9.5	34.6 ± 26.4	30.2 ± 22.5	6.2 ± 2.5	18.9 ± 6.7	26.0 ± 27.8	20.0 ± 8.5
Conventional	22.7 ± 6.0	41.6 ± 14.6	37.7 ± 14.2	6.0 ± 1.0	18.3 ± 6.0	29.0 ± 22.6	29.7 ± 11.7
P-value	< 0.01	0.02	< 0.01	0.19	0.23	0.17	< 0.01

## Discussion

We determined that conservative oxygenation therapy among ICU patients resulted in significantly lower ICU mortality, 28-day mortality, in-hospital mortality, and new-onset non-respiratory organ failure compared to conventional oxygenation therapy. This supports our hypothesis that patients receiving conservative oxygenation therapy result in significantly superior outcomes compared to patients receiving conventional oxygenation therapy. To our knowledge, this is the first systematic review and meta-analysis to evaluate the outcomes of conservative versus conventional oxygenation therapies, specifically among ICU patients.

Suzuki et al. and Girardis et al. were among the analyzed studies that found that conservative oxygenation therapy resulted in lower onset of new infections and non-respiratory organ failure [24,25]. However, there was no statistical difference in the onset of infections after combining the data. On the other hand, conservative oxygenation therapy resulted in significantly lower onset of non-respiratory organ failure. Studies have shown that hyperoxemia reduces systemic oxygen delivery, cerebral blood flow, and cardiac output, while simultaneously increasing reactive oxygen species leading to oxidative stress. These pieces of evidence may explain the results observed in the investigation of non-respiratory organ failure [26-28].

The higher prevalence for hypoxemia among mechanically ventilated patients may be a postulated concern for worse outcomes with conservative oxygenation. However, this investigation with a similar proportion of mechanically ventilated and non-ventilated patients demonstrated better outcomes among patients receiving conservative oxygenation. This may be explained by a higher risk of developing hyperoxia-induced pulmonary toxicity, which has been associated with higher mortality and worse outcomes, particularly among mechanically ventilated patients [29,30].

There are several limitations among the studies included in the review. None of the studies were blinded, causing possible interviewer bias. The heterogeneity of outcome measures used among the studies limited the data analysis to seven outcome measures. Furthermore, there was a slight heterogeneity of SpO2 targets in each study, producing potential bias. The APACHE III scores of the studied patients were also not reported in the study by Girardis et al. Moreover, patients under the conservative oxygenation arm were slightly younger (62.2 ± 3.9 years vs. 64.1 ± 3.5 years) compared to patients under the conventional oxygenation arm. Potential differences in the APACHE III score, as well as age differences, may thus be partially responsible for the observed differences in outcomes. Another possible limitation of this review is that other relevant studies on this topic could have been excluded, despite conducting a systematic search. Future studies can improve through increasing study size and standardizing clinical outcome measures simultaneously. 

## Conclusions

In conclusion, conservative oxygenation therapy could result in significantly lower rates of ICU mortality, 28-day mortality, in-hospital mortality, and new-onset non-respiratory organ failure. Further randomized controlled studies that show clinical outcome improvement in multiple parameters may be worthwhile to assess the true efficacy of this practice.
